# Evaluating Beef Fatty Acid Composition and Lipid Quality in Response to Silage Type and Feeding Intensity During the Finishing Phase

**DOI:** 10.3390/ani16060923

**Published:** 2026-03-15

**Authors:** Zenon Nogalski, Martyna Momot

**Affiliations:** Department of Animal Nutrition, Feed Sciences and Cattle Breeding, Faculty of Animal Bioengineering, University of Warmia and Mazury in Olsztyn, Oczapowskiego 2, 10-719 Olsztyn, Poland

**Keywords:** Holstein–Friesian bulls, total mixed ration, forage-to-concentrate ratio, intramuscular fat, atherogenic index, thrombogenic index, biohydrogenation

## Abstract

The quality of beef fat depends on both intramuscular fat (IMF) content and fatty acid (FA) composition, which can be modulated by finishing diets. This study evaluated the effects of silage type and feeding intensity on IMF deposition, FA profile, desaturase indices, and lipid quality indices in finishing Holstein–Friesian bulls. Thirty-two bulls were assigned to a 2 × 2 factorial design (n = 8/group) and fed total mixed rations for 120 days based on grass silage or maize silage, under intensive (≈50:50 forage:concentrate, DM basis) or semi-intensive feeding (≈70:30). FA composition of *longissimus lumborum* lipids was determined by GC-FID, and lipid quality indices were calculated, including the atherogenic index (AI), thrombogenic index (TI), and the hypocholesterolemic/hypercholesterolemic ratio (h/H). Feeding intensity increased IMF content (*p* = 0.001) and the absolute amounts of major FA classes (g/100 g meat). Silage type primarily affected FA composition by increasing n-3 PUFA and lowering the n-6/n-3 ratio in grass silage diets (*p* = 0.042). Several FAs showed silage type × feeding intensity interactions (*p* < 0.05), indicating that the response to dietary energy supply depended on the forage base. Overall, feeding intensity mainly regulated lipid deposition, whereas silage type modulated the nutritional profile of intramuscular fat.

## Simple Summary

Beef fat quality is important for both consumers and the meat industry as it affects nutritional value and processing properties. The composition of fatty acids in beef can be modified through feeding strategies applied during the finishing period of cattle. In this study, Holstein–Friesian bulls were fed diets differing in silage type (grass or maize silage) and feeding intensity. We examined how these factors influenced the amount of intramuscular fat and the composition of fatty acids in beef. Increasing feeding intensity mainly increased the amount of fat deposited in muscle, while the type of silage primarily affected the fatty acid composition of the fat. Diets based on grass silage resulted in higher proportions of beneficial n-3 fatty acids and a more favourable balance between n-6 and n-3 fatty acids compared with maize silage-based diets. Overall, the results show that combining appropriate silage type with feeding intensity allows producers to influence the nutritional quality of beef fat without markedly changing its technological quality.

## 1. Introduction

In recent years, increasing attention has been paid to the quality of beef fat, not only in terms of sensory attributes but also with regard to its nutritional value and potential implications for human health. This applied interest aligns with growing evidence that feeding strategies and dietary composition can systematically modulate ruminant fat content and fatty acid composition, thereby affecting both consumer-relevant quality attributes and nutritional indices of meat and milk [1,2]. The fatty acid composition of beef, including the proportions of saturated (SFAs), monounsaturated (MUFAs), and polyunsaturated fatty acids (PUFAs), as well as the relationships among these fractions, is considered one of the key indicators of final product quality [3,4]. Particular attention is often given to oleic acid (C18:1 cis-9), which typically represents the dominant monounsaturated fatty acid in beef lipids and commonly accounts for approximately 35–45% of total fatty acids in intramuscular fat [5,6]. From a metabolic perspective, oleic acid is the principal product of stearoyl-CoA desaturase (Δ9-desaturase), which catalyses the conversion of stearic acid (C18:0) to C18:1 cis-9 in animal tissues. However, variations in the proportion of C18:1 cis-9 cannot be interpreted solely as a direct reflection of desaturase activity, because they may also result from differences in dietary fatty acid supply, ruminal biohydrogenation pathways, and the balance between de novo lipogenesis and deposition of pre-formed fatty acids. For this reason, indirect desaturation indices derived from fatty acid ratios (e.g., C18:1/C18:0) are frequently used to provide a more mechanistic interpretation of lipid metabolism in ruminant muscle. Although higher proportions of oleic acid have sometimes been associated with favourable technological or sensory properties of beef, such traits were not evaluated in the present study; therefore, the present work focuses primarily on the metabolic and nutritional interpretation of fatty acid composition. Consequently, there is increasing demand for practical finishing strategies that improve the nutritional quality of beef lipids without compromising technological and sensory attributes.

One of the most important factors shaping the fatty acid profile of beef is the feeding strategy, including the type of forage used and the intensity of fattening. In the present study, ‘feeding (fattening) intensity’ refers to the dietary energy density and the forage-to-concentrate ratio of the total mixed ration (TMR), implemented here as approximately 50:50 (intensive) versus 70:30 (semi-intensive) on a DM basis. It has been demonstrated that diets based on grass silage promote a higher proportion of n-3 fatty acids and a lower n-6/n-3 ratio, whereas maize silage-based diets and a higher proportion of concentrates are more frequently associated with an increased proportion of MUFA, including C18:1 cis-9 [3,7,8,9]. These differences result not only from variations in the lipid composition of feeds but also from interactions between diet, ruminal lipid transformations, and tissue metabolism, which together determine the rate of lipogenesis and the composition of triacylglycerol and phospholipid fractions in muscle.

Feeding intensity and finishing strategies play a crucial role in regulating lipogenic processes and shaping the culinary properties of beef. Studies conducted in Holstein–Friesian bulls have shown that less intensive fattening systems favour a higher proportion of PUFA and a reduced n-6/n-3 ratio in meat, despite a concomitant reduction in growth rate [10]. Increasing dietary energy density has been reported to enhance intramuscular fat (IMF) deposition but simultaneously shift fatty acid proportions towards a higher MUFA content at the expense of the relative contribution of PUFA, a phenomenon often described as the ‘dilution effect’ of phospholipids due to the expansion of the triacylglycerol fraction [11]. Importantly, greater intramuscular fat deposition (marbling) does not automatically translate into improved nutritional quality, because increasing triacylglycerol accumulation may dilute the phospholipid fraction and reduce the relative contribution of PUFA (‘dilution effect’). Therefore, both IMF level and detailed fatty acid profile should be considered when evaluating finishing strategies. Consequently, improvements in marbling do not necessarily translate into parallel improvements in the nutritional quality of fat, and the evaluation of feeding effects should encompass both IMF content and the detailed fatty acid profile, as well as lipid quality indices. Moreover, studies comparing different rations during the finishing period have demonstrated that modifications in the proportion of forage components (e.g., lucerne, maize stover, maize silage) may influence selected sensory attributes, such as muscle fibre tenderness and juiciness, as well as the proportions of specific fatty acid fractions [12]. This highlights the need to simultaneously consider sensory quality and lipid quality within the context of feeding systems applied during the finishing period.

Beef lipid and technological quality are also influenced by the overall production system and management intensity (e.g., feeding level, finishing duration, and slaughter maturity), which jointly affect the rate of fat accretion and the balance between lipid fractions in muscle [13,14]. Feeding intensity and diet formulation during the finishing phase determine the rate of fat deposition and the proportions of lipid fractions within muscle tissue, which may translate into changes in technological meat traits, including tenderness, colour, and water-holding capacity [13,15]. It has been shown that increased slaughter weight and higher dietary energy concentration promote intramuscular fat accumulation but may also lead to alterations in the balance between saturated and polyunsaturated fatty acids and changes in the n-6/n-3 ratio [14,15]. From a practical production perspective, this necessitates the development of feeding strategies during the finishing period that balance production efficiency with quality objectives, encompassing both technological properties and the lipid profile and nutritional value of beef [4,13].

Because consumers primarily purchase and evaluate lean muscle cuts, the present study focuses on intramuscular fat (IMF) in the *longissimus lumborum*. Intramuscular fat determines marbling intensity and contributes to eating quality, but it also represents the principal lipid depot influencing the fatty acid composition of edible muscle tissue. Moreover, IMF affects visual characteristics of meat, including colour appearance and marbling patterns that influence consumer perception and purchasing decisions. For these reasons, intramuscular fat is widely used as a key indicator linking feeding strategies with both the nutritional quality and marketability of ruminant meat products [1,2]. Interpretation of changes in the lipid profile requires consideration not only of IMF content but also of fat deposition site and animal-related factors. Previous studies have demonstrated that fatty acid composition differs among fat depots (intramuscular, intermuscular, subcutaneous, and perirenal) and between sexes (bulls vs. steers), and in some experimental systems, feeding intensity was not the dominant factor compared with fat depot location [16]. This further underscores the importance of using indices that describe metabolic processes (e.g., desaturation indices), rather than relying solely on simple proportions of SFA, MUFA, and PUFA.

Increasing attention has also been devoted to desaturase indices, which serve as indirect indicators of the activity of Δ9-desaturase (stearoyl-CoA desaturase). This enzyme catalyses the conversion of saturated fatty acids, such as C16:0 and C18:0, into their monounsaturated counterparts (C16:1 and C18:1) and is closely associated with the intensity of lipogenic processes and energy metabolism [17,18]. Complementary to this approach are lipid quality indices (e.g., AI, TI, and h/H), which integrate the relative importance of individual fatty acids in terms of atherogenic and thrombogenic risk, as well as potential nutritional benefits [4]. Indirect desaturase indices derived from fatty acid ratios are widely used in meat science studies because they can be calculated from routine GC profiles of post mortem samples, enabling practical, comparable inference on desaturation capacity when direct enzyme activity or gene expression measurements are not available.

In a previous study, Nogalski et al. [19] demonstrated a significant effect of silage type and feeding intensity on carcass traits and intramuscular fat content in fattening cattle. However, that analysis focused primarily on quantitative aspects of fat deposition and did not provide a comprehensive characterisation of fatty acid composition and lipid quality. Since changes in IMF content do not necessarily reflect modifications in fatty acid profile, further evaluation including individual fatty acids, desaturase indices as proxies of lipid metabolism, and composite lipid quality indices is warranted. Although numerous studies have investigated the effects of forage type or concentrate level on beef fatty acids [4,7,13,20], relatively few have simultaneously integrated these parameters within a single factorial design. The present 2 × 2 (silage type × feeding intensity) experiment in finishing Holstein–Friesian bulls therefore addresses this gap by combining quantitative lipid deposition data with a detailed qualitative assessment of fatty acid profile and lipid quality.

Continuation of the research initiated by Nogalski et al. [19] allows this knowledge gap to be addressed by providing data that improve understanding of how manipulation of silage type and feeding intensity modulates (i) the proportions of key fatty acids, (ii) indirect measures of lipogenic enzyme activity, and (iii) integrated lipid quality indices relevant to consumers. The results obtained may contribute to the current body of knowledge and provide practical guidance for optimising feeding strategies for finishing cattle in order to improve the lipid quality of beef, in line with consumer expectations and contemporary trends in animal-derived food production. We hypothesised that: (1) grass silage, compared with maize silage, would increase the proportion of n-3 fatty acids and improve the n-6/n-3 ratio; (2) higher feeding intensity would increase IMF deposition and absolute amounts of fatty acids per unit of meat; and (3) the combined dietary strategy could modulate desaturase indices and composite lipid quality indices.

The aim of this study was to evaluate the effect of silage type and feeding intensity on the detailed fatty acid profile, desaturase activity indices, and lipid quality indices of beef, as a continuation of earlier research focusing on carcass traits and intramuscular fat content.

## 2. Materials and Methods

### 2.1. Animals, Housing, Experimental Design, and Diets

The experiment was conducted in accordance with the feeding and management scheme described by Nogalski et al. [19]. Thirty-two Holstein–Friesian bulls originating from a dairy herd were used. During the rearing period, calves were managed conventionally, and from approximately five months of age they were fattened semi-intensively using total mixed rations (TMRs). At the beginning of the finishing period (approximately 600 days of age; mean body weight about 530 kg), bulls were allocated to experimental treatments using the analogue method to equalise age and body weight among groups. Animals were housed in group pens on deep bedding with free access to water and mineral salt blocks, and each bull was considered an experimental unit. Throughout the experiment, animals remained under veterinary supervision and no health disorders affecting carcass or meat quality were observed. The finishing period lasted 120 days and followed a 2 × 2 factorial design with silage type (TS: grass silage—GS or maize silage—MS) and feeding intensity (FI: intensive—I or semi-intensive—SI) as fixed factors. Four dietary treatments were therefore applied: GS-I, GS-SI, MS-I, and MS-SI (n = 8 bulls per treatment). Bulls entered the finishing phase at approximately 600 days of age, reflecting regional production practice for dairy-origin Holstein–Friesian bulls finished to relatively high slaughter weights.

Animals were fed ad libitum TMR diets formulated according to INRA [21] recommendations to meet the nutrient requirements of finishing bulls. Feeding intensity was modulated by altering dietary energy density through the forage-to-concentrate ratio. In intensive diets the forage-to-concentrate ratio was approximately 50:50 (DM basis), whereas in semi-intensive diets it was approximately 70:30. Crude protein concentration was maintained at a similar level across treatments, while silage type determined the forage base of the diet. The Roughage Intake Control System (Insentec BV, Marknesse, The Netherlands) was used to monitor individual feed intake to ensure accurate feed allocation and dietary control during the experiment. Intake data were recorded for feeding management purposes but were not analysed as response variables in the present study, which focuses on intramuscular fatty acid composition and lipid quality indices. The chemical composition and fatty acid profiles of silages and concentrate components are presented in Table 1, whereas the ingredient composition, chemical composition, and nutritive value of the experimental diets are shown in Table 2.

### 2.2. Feed Sampling and Chemical Analyses

Representative samples of silages and concentrates were collected once a week throughout the experiment. Dry matter, crude protein, ether extract, and ash were determined according to AOAC [22] procedures. Neutral detergent fibre (NDF), acid detergent fibre (ADF), and acid detergent lignin (ADL) were analysed using the detergent method described by Van Soest et al. [23]. Silage pH was measured potentiometrically. Concentrations of lactic, acetic, and butyric acids were determined in aqueous extracts according to the principles described by McDonald et al. [24]. Fatty acid composition of feeds was analysed by gas chromatography after preparation of fatty acid methyl esters (FAMEs) using the modified Peisker method [25].

### 2.3. Slaughter Procedure and Meat Sampling

At the end of the finishing period, bulls were transported to a commercial abattoir and rested for 15–20 h with access to water. All slaughter and post mortem procedures complied with Council Regulation (EC) No. 1099/2009. Samples of the *longissimus lumborum* muscle were excised from chilled carcasses 96 h post mortem. Sampling at 96 h post mortem reflected the commercial chilling and carcass processing schedule, ensuring consistent temperature stabilisation before excision. Samples of the *longissimus lumborum* were collected from the same anatomical location in all carcasses. Samples of the *longissimus lumborum* muscle were collected between the third and fifth lumbar vertebrae (L3–L5). Muscle samples were vacuum-packed, aged at 4 ± 1 °C until day 14 post mortem, and then frozen at −20 °C until analysis.

### 2.4. Intramuscular Fat Content and Fatty Acid Profile

Chemical composition of the *longissimus lumborum* muscle was determined after the ageing period. Intramuscular fat (IMF) content was determined by Soxhlet extraction using a Buechi B-811 extraction system (BÜCHI Labortechnik AG, Flawil, Switzerland) with hexane as the solvent, in accordance with AOAC Method 991.36 9 [22], and the results are presented in Table 3. For fatty acid analysis, muscle samples were ground and homogenised using an Ultra-Turrax homogeniser (Janke & Kunkel, IKA-Werke GmbH & Co. KG, Staufen im Breisgau, Germany), and total lipids were extracted from the homogenised samples using the same Soxhlet procedure. Fatty acid methyl esters (FAMEs) were prepared according to PN-EN ISO 5509 (2001) [26] using the modified Peisker method [25], involving methylation in a methanol–chloroform–H_2_SO_4_ mixture. Fatty acid separation and quantification were performed by gas chromatography with flame ionisation detection (GC-FID) using a Varian CP 3800 gas chromatograph equipped with a split/splitless injector and a CP-Sil 88 capillary column (100 m × 0.25 mm i.d.) (Varian Inc., Palo Alto, CA, USA), in accordance with PN-EN ISO 5508 (1996). Helium was used as the carrier gas. Injector and detector temperatures were set at 260 °C. The oven temperature was programmed from 110 °C to 249 °C. Chromatographic data were processed using the Galaxie Chromatography Data System (Varian Star Chromatography Workstation ver. 6.30; Varian Inc., Palo Alto, CA, USA).

Individual fatty acids were identified by comparing their retention times with those of certified reference standards (Supelco Inc., Bellefonte, PA, USA), analysed under identical chromatographic conditions. The proportion of 31 fatty acids was determined and expressed as a percentage of the total identified fatty acids (Table 4). Fatty acids were grouped into saturated (SFA), monounsaturated (MUFA) and polyunsaturated (PUFA) fractions. In addition, the ratios PUFA/SFA and n-6/n-3 were calculated to characterise the nutritional quality of intramuscular fat. Total lipids were extracted using Soxhlet to ensure exhaustive recovery of intramuscular lipids for consistent GC profiling across treatments; however, we acknowledge that extraction approach and total lipid analysis do not separate neutral lipids from phospholipids, which may limit depot fraction specific interpretation.

Nutritional quality of intramuscular fat was assessed using lipid quality indices calculated from the fatty acid profile (Table 5). The indices values were calculated according to Ulbricht and Southgate [27] and Boukrouh et al. [28]:

$$AI=\frac{C12:0+4\times C14:0+C16:0}{\Sigma MUFA+\Sigma PUFA}$$

$$TI=\frac{C14:0+C16:0+C18:0}{\left(0.5\times \Sigma MUFA)+(0.5\times \Sigma PUFA\;n-6)+(3\times \Sigma PUFA\;n-3)+(\Sigma PUFA\;n-3/\Sigma PUFA\;n-6\right)}$$

The hypocholesterolemic/hypercholesterolemic ratio (h/H) was calculated as:

h/H = (C18:1 cis-9 + C18:2 n-6 + C20:4 n-6 + C18:3 n-3 + C20:5 n-3 + C22:5 n-3 + C22:6 n-3)/(C14:0 + C16:0).

In addition, EPA + DHA, LA/ALA, desirable fatty acids (DFAs), and Δ9-desaturase indices were calculated as follows:

EPA + DHA = C20:5 n-3 + C22:6 n-3; LA/ALA = C18:2 n-6/C18:3 n-3; DFA = (ΣMUFA + ΣPUFA + C18:0)

These indices were used to evaluate the potential impact of dietary treatments on the nutritional quality of beef fat.

### 2.5. Statistical Analysis

Data were analysed using a two-factorial least squares model including the fixed effects of silage type (TS), feeding intensity (FI), and their interaction (TS × FI):

$$Y_{ijk}=\mu +TS_{i}+FI_{j}+(TS\times FI{)}_{ij}+e_{ijk}$$

where $Y_{ijk}$ represents the analysed trait, μ is the overall mean, $TS_{i}$ is the effect of silage type, $FI_{j}$ is the effect of feeding intensity, $\left(TS\times FI{)}_{ij}\right.$ is the interaction between silage type and feeding intensity, and $e_{ijk}$ is the random residual error.

Model residuals were checked for normality using the Shapiro–Wilk test and visual inspection of Q–Q plots and for homogeneity of variance using Levene’s test. When significant main effects or interactions were detected (*p* < 0.05), pairwise comparisons were performed using Tukey’s post hoc test.

Results are presented as least squares means (LSMeans) with their corresponding standard error of the mean (SEM). Differences were considered statistically significant at *p* < 0.05 and highly significant at *p* < 0.01. All statistical analyses were conducted using Statistica software (Data Analysis Software System, version 13.3; TIBCO Software Inc., Tulsa, OK, USA).

### 2.6. Growth Performance and Carcass Fatness (Context Variables)

Growth performance and carcass characteristics included in Section 3.1 were recorded following the protocol described in detail by Nogalski et al. [19]. Briefly, body weight was measured at the beginning and at the end of the 120-day finishing period, and average daily gain was calculated accordingly. After slaughter, carcass fatness was assessed under commercial abattoir conditions as reported previously [19]. These variables are presented only to contextualize intramuscular fat deposition and fatty acid results, whereas the main analytical focus of the present study is the intramuscular lipid profile and derived indices.

## 3. Results

### 3.1. Growth Performance and Carcass Fatness

Results concerning growth performance and carcass characteristics obtained in the first part of this experiment were reported previously by Nogalski et al. [19]. Briefly, finishing Holstein–Friesian bulls reached final body weights of approximately 620–690 kg, with higher values observed under intensive feeding. Average daily gains ranged from about 0.90 to 1.25 kg day^−1^ and were significantly affected by feeding intensity. Carcass fatness differed among dietary treatments, with higher fatness scores observed in intensively fed bulls and in animals receiving maize silage-based diets. These results provide the growth and carcass background for the present evaluation of intramuscular fat content and fatty acid composition.

### 3.2. Intramuscular Fat Content and Individual Fatty Acid Composition

The intramuscular fat (IMF) content of the *longissimus lumborum* muscle was affected by feeding intensity (*p* = 0.001), whereas silage type had no significant effect (*p* = 0.405) (Table 3). Higher IMF levels were observed in intensively fed bulls compared with those fed semi-intensively. No significant TS × FI interaction was detected for IMF content (*p* = 0.886).

The proportions of several individual fatty acids were influenced by silage type, feeding intensity, or their interaction (Table 3). Feeding intensity affected the proportions of C14:0 (*p* = 0.025), C15:0 (*p* = 0.018), C18:1 c11 (*p* = 0.013), C18:2 n-6 (*p* = 0.022), and long-chain n-3 fatty acids, including DPA (*p* = 0.003) and DHA (*p* = 0.040). Silage type influenced C18:0 (*p* = 0.016), C18:1 c11 (*p* = 0.048), EPA (*p* = 0.022), and DPA (*p* = 0.027). Significant TS × FI interactions were observed for C15:0 (*p* = 0.041), C17:0 (*p* = 0.027), C18:3 n-3 (*p* = 0.018), and DPA (*p* = 0.026).

Fatty acid group composition was affected by both dietary factors (Table 4). Feeding intensity significantly influenced the proportions of ΣSFA, ΣPUFA, Σn-6, Σn-3, and the PUFA/SFA ratio. Silage type significantly affected Σn-3 content and the n-6/n-3 ratio. A significant TS × FI interaction was detected for Σn-3 and PUFA/SFA, indicating differential responses of these parameters to feeding intensity depending on silage type.

### 3.3. Nutritional Quality and Desaturase Indices

Nutritional quality indices calculated from the fatty acid profile are presented in Table 5. Feeding intensity affected AI (*p* = 0.041) and the h/H ratio (*p* = 0.023), whereas silage type influenced TI (*p* = 0.043) and the desaturase index 18 (*p* = 0.046). No significant TS × FI interactions were observed for lipid quality indices or desaturase indices.

### 3.4. Fatty Acids Expressed per 100 g of Meat and Interaction Effects

When expressed as g per 100 g of meat, selected fatty acids were influenced primarily by feeding intensity, reflecting differences in IMF content (Table 6). To facilitate interpretation of the significant TS × FI interactions detected in the relative fatty acid composition, selected interaction effects are presented separately in Table 7. These results illustrate that the combined effects of silage type and feeding intensity modulated the deposition of specific fatty acids in intramuscular fat.

## 4. Discussion

As background, the same experimental population showed higher final body weight and average daily gain under intensive feeding, whereas carcass fatness was higher in intensively fed bulls and in animals receiving maize silage-based diets, as reported previously [19]. These responses are consistent with the present IMF results, because greater dietary energy supply and advanced carcass fatness typically promote intramuscular lipid accretion and increase the absolute deposition of fatty acids per unit of meat. Therefore, growth performance and carcass fatness provide biological context for interpreting the lipid outcomes, supporting the conclusion that feeding intensity primarily regulates lipid deposition, whereas silage type mainly modulates fatty acid composition.

Feeding intensity increased IMF content and, consequently, the absolute amounts of major fatty acid classes expressed per 100 g of meat (Table 6), whereas silage type mainly modulated the relative fatty acid profile, particularly Σn-3 and the *n*-6/*n*-3 ratio (Table 3, Table 4 and Table 5). These patterns provide the basis for the mechanistic interpretation below. The observed differences in intramuscular fatty acid composition resulted from the combined effects of dietary energy supply, forage type, and ruminal metabolism, which together regulate lipid deposition and post-absorptive modification in ruminant tissues [29,30]. Feeding intensity primarily influenced intramuscular fat content and the absolute deposition of fatty acids, whereas silage type, and its interaction with feeding intensity, modulated the qualitative fatty acid profile (Table 3, Table 4, Table 5, Table 6 and Table 7).

The working hypothesis assumed that feeding intensity would mainly determine the extent of lipid deposition, while silage type would influence fatty acid composition and lipid quality indices through differences in precursor supply and ruminal biohydrogenation pathways. The results largely confirm this assumption; however, several significant interactions indicate that dietary energy density and forage base operate in a complementary rather than independent manner.

The higher intramuscular fat content observed under intensive feeding can be explained by increased dietary energy availability exceeding the requirements for maintenance and lean tissue growth, thereby favouring lipogenesis in intramuscular adipocytes [15,31,32]. The increase in IMF under intensive feeding was accompanied by higher absolute FA amounts (g/100 g meat), while relative proportions (% of total FAs) may shift due to the ‘dilution effect’, whereby expanding triacylglycerol deposition reduces the relative contribution of membrane phospholipids that are richer in PUFA. In ruminants, de novo synthesis of fatty acids in adipose tissue relies mainly on acetate and β-hydroxybutyrate produced during ruminal fermentation, and diets with a higher concentrate proportion typically enhance insulin secretion and lipogenic enzyme activity [15,33]. Consequently, increased intramuscular fat content under intensive feeding is commonly associated with higher absolute amounts of fatty acids expressed per unit of meat, as observed in the present study (Table 6), and has been reported previously by Morales et al. [4], Nogalski et al. [5], and Dykier et al. [20]. The increase in intramuscular fat under intensive feeding is consistent with previous studies showing that higher dietary energy density enhances lipogenesis in ruminant muscle, particularly via increased acetate availability and greater insulin-mediated stimulation of lipid synthesis. Similar responses have been reported in dairy-type bulls and beef breeds under high-concentrate finishing systems [5,10,19]. Increased IMF is often accompanied by a relative dilution of PUFAs due to the preferential deposition of de novo synthesized SFAs and MUFAs, a phenomenon frequently described as the ‘dilution effect’ in ruminant lipid metabolism.

Differences in odd-chain fatty acids (C15:0 and C17:0), together with significant TS × FI interactions, likely reflect alterations in ruminal microbial synthesis. Among odd-chain fatty acids, C15:0 showed a clear TS × FI interaction (Table 7), whereas C17:0 responded more modestly. Such shifts are consistent with diet-driven changes in rumen fermentation and microbial lipid flow, often contrasting higher-concentrate versus forage-based finishing conditions. Odd-chain fatty acids originate predominantly from microbial cell membranes and are incorporated into animal tissues following intestinal absorption of microbial lipids [30,34]. Changes in forage type and feeding intensity modify ruminal pH, passage rate, and microbial community structure, thereby affecting the contribution of odd-chain fatty acids to intramuscular fat [3,4].

Silage type influenced the proportions of long-chain polyunsaturated fatty acids, particularly within the n-3 family, which can be mechanistically linked to differences in precursor supply and ruminal biohydrogenation. Grass silage typically contains higher concentrations of α-linolenic acid (C18:3 n-3), which influences ruminal biohydrogenation dynamics and post-absorptive fatty acid availability [3,7,35]. Although extensive biohydrogenation of unsaturated fatty acids occurs in the rumen, a fraction escapes hydrogenation and is absorbed in the small intestine [29,30]. Subsequently, α-linolenic acid may serve as a substrate for elongation and desaturation processes in animal tissues, leading to the formation of long-chain n-3 fatty acids such as EPA and DPA [17,35]. Although EPA and DPA increased under grass silage, their absolute proportions remained low, which is consistent with the limited conversion efficiency of α-linolenic acid to long-chain n-3 fatty acids in ruminants. In the present study, EPA ranged from approximately 0.07 to 0.10% of total fatty acids, whereas DPA varied between about 0.15 and 0.24% of total fatty acids (Table 3), indicating that although statistically significant differences were detected, the absolute magnitude of change remained relatively small. Nevertheless, even small relative increases in these fatty acids contribute to lowering the n-6/n-3 ratio, a commonly used indicator of the nutritional quality of meat lipids [36]. The observed effects of silage type on n-3 fatty acids and the n-6/n-3 ratio are therefore consistent with established dietary modulation of fatty acid precursors.

The significant interactions between silage type and feeding intensity for selected fatty acids and fatty acid groups indicate that increased energy supply modifies fatty acid deposition differently depending on the forage base of the diet (Table 3, Table 4 and Table 7). A clear example of TS × FI interaction was observed for Σn-3, where GS-SI showed the highest value among treatment combinations (Table 7). This suggests that the effect of added concentrate/energy depends on forage base, likely through differences in ruminal pH, passage rate, and biohydrogenation pathways that determine the escape of unsaturated FA intermediates. Similar interaction patterns have been reported by Morales et al. [4] and Torrecilhas et al. [13], suggesting that feeding intensity does not uniformly affect fatty acid metabolism but interacts with forage-derived lipid supply and ruminal fermentation characteristics. In line with this concept, Momot et al. [10] demonstrated that changes in feeding intensity alone were sufficient to alter the proportions of PUFA and the n-6/n-3 ratio in beef, highlighting the sensitivity of fatty acid deposition to dietary energy supply even under a constant forage base.

The effect of silage type on n-3 fatty acids and the n-6/n-3 ratio can be mechanistically linked to differences in fatty acid composition of the forage base. Grass silage typically contains higher concentrations of α-linolenic acid (C18:3 n-3), whereas maize silage provides more linoleic acid (C18:2 n-6) [3,7]. Although extensive ruminal biohydrogenation of unsaturated fatty acids limits their direct transfer to tissues, a proportion escapes hydrogenation [29,30,35] and may serve as a substrate for elongation and desaturation processes in muscle, leading to the formation of long-chain n-3 PUFAs such as EPAs and DPAs [17,33]. Consequently, dietary forage type may indirectly influence the deposition of long-chain n-3 PUFAs even under high-concentrate finishing systems.

Despite changes in individual fatty acids, nutritional quality indices such as AI, TI, and h/H were only moderately affected and showed no significant interactions. Changes in desaturase indices further suggest that dietary treatments may influence endogenous lipid metabolism beyond simple precursor supply. The Δ9-desaturase index reflects stearoyl-CoA desaturase activity, which catalyses the conversion of saturated fatty acids (e.g., C16:0 and C18:0) into their monounsaturated counterparts and plays a key role in determining both sensory attributes and nutritional properties of beef lipids [15,17]. Variations observed in desaturase index 18 in the present study indicate that silage type may modulate lipogenic enzyme activity, potentially through differences in energy balance, substrate availability, and hormonal regulation. Such metabolic adjustments complement the direct dietary effects on fatty acid precursor supply and help explain why relative changes in individual fatty acids may not always translate proportionally into composite lipid quality indices. This indicates that compensatory shifts among different fatty acid classes may stabilise composite lipid quality indices, even when individual fatty acids respond to dietary treatments [6,37]. In beef and other ruminant meats, reported values of AI and TI generally range from approximately 0.5 to 1.0 and from 1.0 to 2.0, respectively, whereas h/H ratios commonly range between about 1.5 and 2.5 depending on diet and fat depot. In the present study, AI ranged from 0.63 to 0.69, TI from 1.40 to 1.53, and h/H from 1.52 to 1.67 (Table 5), indicating that all values fall within the typical ranges reported for ruminant meat lipids. Although feeding intensity and silage type produced statistically significant differences in some indices, the absolute magnitude of change was relatively small (e.g., ΔAI ≈ 0.06 and Δh/H ≈ 0.15), suggesting that the practical nutritional impact of these variations is likely limited. Nevertheless, such indices remain useful for comparing dietary strategies and identifying directional changes in lipid quality of beef.

Overall, the present results confirm that feeding intensity predominantly regulates the extent of intramuscular lipid deposition, whereas silage type determines the qualitative fatty acid profile through differences in precursor supply and ruminal biohydrogenation pathways. The interaction between these factors highlights the complexity of nutritional control over lipid metabolism in beef cattle, as previously described in studies focusing on forage-based and mixed feeding systems [9,29]. From a practical standpoint, combining forage base with an appropriate feeding intensity provides a feasible lever to shape beef lipid quality: grass silage supports a more favourable n-3 profile, whereas higher feeding intensity primarily increases IMF and absolute fatty acid deposition.

From a practical production perspective, the present results are most relevant for dairy-origin Holstein–Friesian bulls finished under semi-intensive or intensive TMR systems typical of many European beef production chains. In this context, manipulation of the forage base and feeding intensity provides a practical nutritional tool for modulating intramuscular lipid deposition and fatty acid composition. In the present study, increasing feeding intensity raised intramuscular fat from approximately 2.26 to 3.50%, whereas grass silage diets increased the proportion of n-3 fatty acids from about 0.88 to 1.09% of total fatty acids and reduced the n-6/n-3 ratio from about 4.84 to 4.19. Although these changes were statistically significant, their absolute magnitude remains relatively modest. Consequently, while such dietary strategies may contribute to incremental improvements in the nutritional profile of beef lipids within production systems, they are unlikely on their own to support specific nutritional claims at the consumer level.

This study also has several limitations that should be considered when interpreting the results. These include the use of a single dairy breed (Holstein–Friesian), the focus on one intramuscular depot (*longissimus lumborum*), and the analysis of total lipids without separation of neutral and polar lipid fractions. Future research integrating intake data, additional muscles, and lipid fraction analysis could provide deeper insight into the metabolic mechanisms underlying diet-induced changes in beef lipid composition.

## 5. Conclusions

The results of this study demonstrate that both feeding intensity and silage type significantly influence the fatty acid composition of intramuscular fat in finishing Holstein–Friesian bulls. Feeding intensity was the primary factor determining the amount of intramuscular fat and the absolute deposition of fatty acids in beef, whereas silage type mainly shaped the qualitative fatty acid profile, particularly with respect to n-3 fatty acids and the n-6/n-3 ratio.

The interaction between silage type and feeding intensity further modulated selected fatty acids and fatty acid groups, indicating that the response to increased dietary energy supply depended on the forage base of the diet. These findings highlight the importance of considering combined dietary strategies rather than isolated nutritional factors when aiming to modify the lipid composition of beef.

Despite changes observed in individual fatty acids, nutritional quality indices showed relatively limited variation, suggesting a degree of stability in overall lipid quality of beef under different feeding regimes. Overall, the present study confirms that targeted manipulation of feeding intensity and silage type can be used to influence both the quantity and composition of intramuscular fat, providing a nutritional basis for optimising beef fatty acid profiles under practical finishing conditions.

## Figures and Tables

**Table 1:** Chemical and fatty acid composition of experimental fodders (mean ± standard error of mean).

Specification	Grass Silage	Maize Silage	Triticale	Rapeseed Meal
Chemical composition (g∙kg^−1^ DM) of experimental fodders				
Dry matter g∙kg^−1^	285	322	875	878
Organic matter	906	967	966	921
Crude protein	121	88.9	122	383
NDF	536	337	162	298
ADF	314	196	41	212
ADL	25.5	12.7	-	-
NFC	194	508	629	237
pH	4.23	3.56		
Lactic acid	43.6	27.5		
Acetic acid	12.5	6.6		
Butyric acid	0.09	0.08		
N-NH_3_ (g kg^−1^ TN)	75.9	33.9		
UFV	0.86	0.85	1.19	1.03
PDIN	83	50	85	254
PDIE	72	68	98	162
Fatty acid profile (g/100 g fatty acids)				
C14:0	0.16 ± 0.01	1.77 ± 0.07		
C16:0	13.99 ± 0.19	22.65 ± 0.24		
C18:0	1.98 ± 0.08	3.09 ± 0.11		
C18:1 n-9 (OA)	22.65 ± 0.09	6.11 ± 0.09		
C18:2 n-6 (LA)	50.78 ± 0.14	24.32 ± 0.12		
C18:3 n-3 (LNA)	8.21 ± 0.09	39.25 ± 0.15		

NDF—neutral detergent fibre; ADF—acid detergent fibre; ADL—acid detergent lignin; NFC—non-fibre carbohydrates; N-NH_3_—ammonia nitrogen; TN—total nitrogen; UFV—meat production units; PDIN—protein digested in the small intestine depending on rumen-degraded protein; PDIE—protein digested in the small intestine depending on rumen-fermented organic matter; OA—oleic acid; LA—linoleic acid; LNA—linolenic acid.

**Table 2:** Ingredients (% DM) and chemical composition of diets.

Specification	GS-I	GS-SI	MS-I	MS-SI
Grass silage	50	70		
Maize silage			50	70
Triticale grain	47	27	41	18
Rapeseed meal	3	3	9	12
Dry matter (g/kg fresh)	580.09	462.09	598.77	488.26
In g/kg DM				
Organic matter	934.65	922.65	962.45	961.3
Crude protein	129.33	129.13	128.94	130.15
NDF	353.08	427.88	261.74	300.82
ADF	182.63	237.23	133.89	170.02
NFC	399.74	312.74	533.22	497.26
UFV	1.02	0.95	1.01	0.93
PDIN	89.07	88.67	82.71	80.78
PDIE	86.92	82.71	87.76	84.68

GS-I—grass silage intensive; GS-SI—grass silage semi-intensive; MS-I—maize silage intensive; MS-SI—maize silage semi-intensive; NDF—neutral detergent fibre; ADF—acid detergent fibre; NFC—non-fibre carbohydrates; UFV—meat production units; PDIN—protein digested in the small intestine depending on rumen-degraded protein; PDIE—protein digested in the small intestine depending on rumen-fermented organic matter.

**Table 3:** Individual fatty acid composition (% of total identified fatty acids).

Fatty Acid (% Total FA)	Type of Silage (TS)		Fattening Intensity (FI)		SEM	*p*-Value		
	Grass Silage	Maize Silage	Intensive	Semi-Intensive		TS	FI	TS × FI
Intramuscular fat (%)	2.74	3.01	3.50	2.26	0.189	0.405	0.001	0.886
C14:0	2.65	2.56	2.77	2.45	0.076	0.731	0.025	0.272
C15:0	0.37	0.42	0.43	0.37	0.013	0.027	0.018	0.041
C16:0	25.68	25.51	26.00	25.19	0.267	0.754	0.146	0.777
C17:0	0.98	1.08	1.05	1.00	0.033	0.108	0.355	0.027
C18:0	13.86	16.00	15.62	14.22	0.485	0.016	0.111	0.275
C16:1 c9	4.02	3.53	3.73	3.83	0.190	0.191	0.784	0.068
C18:1 c9 (oleic)	38.36	37.95	37.80	38.51	0.602	0.732	0.559	0.102
C18:1 c11	1.82	1.61	1.58	1.85	0.057	0.048	0.013	0.525
C18:1 t10 (vaccenic)	2.00	2.08	2.23	1.83	0.151	0.624	0.162	0.204
C18:2 n-6 (LA)	3.81	3.62	3.26	4.14	0.204	0.429	0.022	0.065
C18:3 n-3 (ALA)	0.55	0.47	0.49	0.53	0.022	0.063	0.283	0.018
C20:4 n-6 (ARA)	0.83	0.60	0.48	0.94	0.085	0.122	0.003	0.063
CLA c9.t11	0.41	0.39	0.41	0.39	0.010	0.470	0.145	0.227
EPA (C20:5 n-3)	0.10	0.07	0.07	0.10	0.008	0.022	0.126	0.236
DPA (C22:5 n-3)	0.23	0.16	0.15	0.24	0.016	0.027	0.003	0.026
DHA (C22:6 n-3)	0.03	0.03	0.02	0.03	0.001	0.141	0.040	0.091

Values are presented as the mean ± SEM. LA—linoleic acid; ALA—α-linolenic acid; ARA—arachidonic acid; CLA—conjugated linoleic acid; EPA—eicosapentaenoic acid; DPA—docosapentaenoic acid; DHA—docosahexaenoic acid.

**Table 4:** Fatty acid groups and ratios (% of total fatty acids).

Parameter (% Total FA)	Type of Silage (TS)		Fattening Intensity (FI)		SEM	*p*-Value		
	Grass Silage	Maize Silage	Intensive	Semi-Intensive		TS	FI	TS × FI
ΣSFA	44.15	46.31	46.60	43.88	0.629	0.076	0.026	0.430
ΣMUFA	49.36	47.68	47.86	49.19	0.703	0.480	0.636	0.112
ΣPUFA	6.56	6.04	5.55	7.02	0.373	0.389	0.010	0.057
Σn-6	4.57	4.22	3.75	5.01	0.267	0.561	0.015	0.068
Σn-3	1.09	0.88	0.87	1.10	0.050	0.023	0.015	0.029
n-6/n-3	4.19	4.84	4.44	4.57	0.157	0.042	0.694	0.615
PUFA/SFA	0.15	0.13	0.12	0.16	0.007	0.109	0.001	0.056
MUFA/SFA	1.13	1.04	1.04	1.13	0.030	0.270	0.222	0.171
EPA+DHA	0.12	0.11	0.10	0.12	0.009	0.225	0.186	0.447

Values are presented as the mean ± SEM. ΣSFA—sum of saturated fatty acids; ΣMUFA—sum of monounsaturated fatty acids; ΣPUFA—sum of polyunsaturated fatty acids; Σn-6—sum of n-6 polyunsaturated fatty acids; Σn-3—sum of n-3 polyunsaturated fatty acids.

**Table 5:** Nutritional quality indices (calculated from % FA).

Index	Type of Silage (TS)		Fattening Intensity (FI)		SEM	*p*-Value		
	Grass Silage	Maize Silage	Intensive	Semi-Intensive		TS	FI	TS × FI
AI	0.66	0.67	0.63	0.69	0.016	0.838	0.041	0.191
TI	1.40	1.53	1.41	1.51	0.038	0.043	0.176	0.474
h/H	1.61	1.58	1.52	1.67	0.032	0.602	0.023	0.932
Desaturase index 16	0.14	0.12	0.12	0.14	0.006	0.256	0.539	0.091
Desaturase index 18	0.74	0.70	0.71	0.73	0.009	0.046	0.160	0.214

Values are presented as the mean ± SEM. AI—atherogenic index; TI—thrombogenic index; h/H—hypocholesterolemic to hypercholesterolemic fatty acids ratio.

**Table 6:** Selected fatty acids expressed as g per 100 g of meat (calculated from IMF and % FA).

Fatty Acid (g/100 g Meat)	Type of Silage (TS)		Fattening Intensity (FI)		SEM	*p*-Value		
	Grass Silage	Maize Silage	Intensive	Semi-Intensive		TS	FI	TS × FI
SFAs	1.12	1.31	1.52	0.92	0.084	0.222	0.000	0.620
MUFAs	1.23	1.32	1.56	1.01	0.085	0.599	0.001	0.706
PUFAs	0.16	0.17	0.18	0.15	0.012	0.943	0.334	0.183
CLA	0.010	0.011	0.013	0.008	0.001	0.767	0.000	0.752
Σn-3	0.027	0.024	0.028	0.023	0.002	0.296	0.370	0.150
Σn-6	0.011	0.012	0.012	0.011	0.001	0.993	0.673	0.145
EPA + DHA	0.003	0.003	0.003	0.003	0.000	0.892	0.272	0.860

Values are presented as the mean ± SEM. SFAs—saturated fatty acids; MUFAs—monounsaturated fatty acids; PUFAs—polyunsaturated fatty acids; CLA—conjugated linoleic acid; Σn-3—sum of n-3 polyunsaturated fatty acids; Σn-6—sum of n-6 polyunsaturated fatty acids; EPA+DHA—sum of eicosapentaenoic and docosahexaenoic acids.

**Table 7:** Effects of silage type and feeding intensity combinations on selected fatty acid traits of the *longissimus lumborum* muscle.

Fatty Acid (% Total FA)	GS-I	GS-SI	MS-I	MS-SI	SEM	*p*-Value
C15:0	0.38 ^b^	0.37 ^b^	0.47 ^a^	0.38 ^b^	0.013	0.041
C17:0	0.94 ^b^	1.01 ^ab^	1.17 ^a^	0.98 ^ab^	0.033	0.027
C18:3 n-3 (ALA)	0.48 ^ab^	0.61 ^a^	0.51 ^ab^	0.44 ^b^	0.022	0.018
DPA (C22:5 n-3)	0.14 ^b^	0.30 ^a^	0.15 ^b^	0.18 ^b^	0.016	0.026
Σn-3	0.88 ^b^	1.28 ^a^	0.86 ^b^	0.90 ^b^	0.050	0.029

Values are presented as the mean ± SEM. GS-I, grass silage with intensive feeding; GS-SI, grass silage with semi-intensive feeding; MS-I, maize silage with intensive feeding; MS-SI, maize silage with semi-intensive feeding. Different letters within a row indicate significant differences between treatment combinations at *p* < 0.05 (Tukey’s post hoc test). TS, silage type; FI, feeding intensity; ALA—α-linolenic acid; DPA—docosapentaenoic acid.

## Data Availability

The data presented in this study are available from the corresponding author upon reasonable request.
